# Diversity of CRISPR-Cas type II-A systems in *Streptococcus anginosus*

**DOI:** 10.3389/fmicb.2023.1188671

**Published:** 2023-06-15

**Authors:** Richard Bauer, Dorina Haider, Aline Grempels, Rebecca Roscher, Stefanie Mauerer, Barbara Spellerberg

**Affiliations:** Institute of Medical Microbiology and Hygiene, University Medical Center, Ulm, Germany,

**Keywords:** *Streptococcus anginosus*, CRISPR-Cas, classification, phylogenetic analysis, CSN2 gene

## Abstract

*Streptococcus anginosus* is a commensal Streptococcal species that is often associated with invasive bacterial infections. However, little is known about its molecular genetic background. Many Streptococcal species, including *S. anginosus*, harbor clustered regularly interspaced short palindromic repeats (CRISPR)-Cas systems. A CRISPR-Cas type II-A system as well as a type II-C system have been reported for this species. To characterize the CRISPR-Cas type II systems of *S. anginosus* in more detail, we conducted a phylogenetic analysis of Cas9 sequences from CRISPR-Cas type II systems with a special focus on streptococci and *S. anginosus*. In addition, a phylogenetic analysis of *S. anginosus* strains based on housekeeping genes included in MLST analysis, was performed. All analyzed Cas9 sequences of *S. anginosus* clustered with the Cas9 sequences of CRISPR type II-A systems, including the Cas9 sequences of *S. anginosus* strains reported to harbor a type II-C system. The Cas9 genes of the CRISPR-Cas type II-C systems of other bacterial species separated into a different cluster. Moreover, analyzing the CRISPR loci found in *S. anginosus*, two distinct *csn2* genes could be detected, a short form showing high similarity to the canonical form of the *csn2* gene present in *S. pyogenes*. The second CRISPR type II locus of *S. anginosus* contained a longer variant of *csn2* with close similarities to a *csn2* gene that has previously been described in *Streptococcus thermophilus.* Since CRISPR-Cas type II-C systems do not contain a *csn2* gene, the *S. anginosus* strains reported to have a CRISPR-Cas type II-C system appear to carry a variation of CRISPR-Cas type II-A harboring a long variant of *csn2*.

## Introduction

Bacteria defend themselves against phage associated infection or other invading genetic elements (Barrangou and Marraffini, 2014). In this context the Clustered Regularly Interspaced Short Palindromic Repeats (CRISPR) system has been described as a bacterial immunity system (Bernheim and Sorek, 2020). It contains distinct CRISPR-associated (cas) genes and a CRISPR array composed of unique spacer sequences interspersed with short repeats (Koonin and Makarova, 2009; Deveau et al., 2010; Le Rhun et al., 2019). The principle of the CRISPR-Cas system is based on the integration of new spacers derived from foreign genetic elements into the CRISPR array, that is further transcribed and processed into CRISPR RNAs (crRNAs), consisting of a part of the repeat and the spacer (Brouns et al., 2008; Wiedenheft et al., 2012; Makarova et al., 2015; Wei et al., 2015). Mature crRNAs guide Cas proteins to target sequences on invading nucleic acid mediating specific cleavage (Marraffini and Sontheimer, 2008; Garneau et al., 2010; Hille et al., 2018).

Based upon *cas* gene content, repeat sequence and the organization of the CRISPR loci, CRISPR-Cas systems are currently divided into two classes including three main types each, ranging from type I to VI (class 1 comprising type I, III and IV; class 2 comprising type II, V, VI) and numerous subtypes (Shmakov et al., 2015; Le Rhun et al., 2019; Makarova et al., 2020). As opposed to class 1 systems employing multi-subunit Cas protein complexes, effector modules of class 2 systems only rely on a single Cas protein as corresponding type II systems with its prominent signature gene *cas9* (Sapranauskas et al., 2011; Koonin et al., 2017; Le Rhun et al., 2019). In type II systems the size of the *cas9* gene and the presence or absence of subtype-specific proteins besides Cas1, Cas2 and Cas9 are criterions for further subdivision into subtypes (II-A, II-C1, II-C2; Makarova et al., 2020). These type II systems are highly represented among pathogens (Fonfara et al., 2014; Louwen et al., 2014). While type II-A systems are characterized by the presence of a Csn2 protein, type II-C does not harbor any accessory protein (Mir et al., 2018; Makarova et al., 2020). Like Cas1 and Cas2, Csn2 is assumed to participate in spacer acquisition during the adaptation stage of CRISPR-mediated immunity (Heler et al., 2015; Wei et al., 2015).

Nearly half of all bacteria are equipped with at least one CRISPR-Cas system (Grissa et al., 2007). In streptococci all three major CRISPR types are represented with type I-C, type II-A and type III-A being the most frequently found subtypes (Louwen et al., 2014), whereas *S. pneumoniae* naturally lacks CRISPR-Cas loci (Bikard et al., 2012). Genome analysis of the *Streptococcus anginosus* group (SAG) revealed 7 of the 18 analyzed strains possessing a CRISPR-Cas system with most strains containing one CRISPR locus, typically a type II-A system (Olson et al., 2013). Type II CRISPR-Cas systems are widely distributed among a variety of different species, with type II-A representing the most commonly found subtype (Chylinski et al., 2014; Louwen et al., 2014). The type II-A system of *S. pyogenes* is very well studied and its Cas9 nuclease is extensively repurposed for genome engineering (Deltcheva et al., 2011; Jinek et al., 2012; Cebrian-Serrano and Davies, 2017). Type II-C CRISPR-Cas systems are the simplest type II systems regarding their structure and while nearly half of the multitude of Cas9 proteins discovered so far are part of the type II-C subtype, they have been sparsely investigated (Mir et al., 2018). Nevertheless, several type II-C systems in Streptococci were published and identified in public CRISPR databases as well (Gong et al., 2019; Lemaire et al., 2022). Considering phylogeny of Cas9, type II-A systems are assumed to be a derivative of type II-C, with the *csn2* gene acquired by type II-A ancestors, since the type II-A branch is embedded within type II-C (Chylinski et al., 2014).

*Streptococcus anginosus* is primarily a commensal of mucosal membranes colonizing many areas of the human body including the oral cavity, the gastrointestinal and the urogenital tract (Whiley et al., 1992; Paster et al., 2001; Facklam, 2002; Pilarczyk-Zurek et al., 2022). Together with the closely related species *Streptococcus constellatus* and *Streptococcus intermedius* it belongs to the SAG (Whiley and Beighton, 1991; Jensen et al., 2013). Bacteria of the SAG can frequently be isolated from blood cultures, abscesses, the respiratory tract of cystic fibrosis patients and have recently been associated with gastric cancer (Parkins et al., 2008; Grinwis et al., 2010; Mukae et al., 2016; Kobo et al., 2017; Zhou et al., 2022). Often overlooked in the past, *S. anginosus* has been increasingly identified in invasive infections during the last years emphasizing its clinical importance as an emerging bacterial pathogen (Laupland et al., 2006; Reissmann et al., 2010; Siegman-Igra et al., 2012). Indeed, the incidence rate of SAG infections (8.65/100,000) even exceeds the combined incidence rates of group A and B streptococci in population-based surveillance studies (Laupland et al., 2006; Jiang et al., 2020).

We previously reported on the relationship between CRISPR-Cas systems and the presence of the ß-hemolysin gene cluster in *Streptococcus anginosus* (Bauer et al., 2020). Similar to other human bacterial pathogens some *S. anginosus* strains harbor CRISPR-Cas type II systems (Olson et al., 2013; Louwen et al., 2014). To get a deeper insight into the CRISPR-Cas type II systems present in the species *S. anginosus* we performed a phylogenetic analysis of *S. anginosus* strains in our strain collection and publicly available whole genome sequences with a special focus on strains that were labeled as harboring a CRISPR-Cas type II-C locus.

## Methods

### Strain collection

Clinical isolates of *S. anginosus* strains originated from the University hospital Aachen and Ulm as described previously (Bauer et al., 2020). Streptococci were routinely grown on sheep blood agar plates (TSA + SB, Oxoid, Basingstoke, UK) at 37°C in a 5% CO_2_ atmosphere. Liquid culture was performed in THY broth (Todd-Hewitt Broth [Oxoid] supplemented with 0.5% yeast extract [BD, Miami]). Strains used in this study are listed in Supplementary Table S1.

### Molecular methods

Bacterial genomic DNA was obtained according to manufacturer’s instructions of standard commercial kits (GenElute^™^ Bacterial Genomic DNA Kits, Sigma-Aldrich, St. Louis, United States). For Polymerase Chain Reaction (PCR) Taq polymerase (Roche, Mannheim, Germany) was used with an initial denaturation step of 3 min at 94°C, 30 amplification cycles of 1 min at 94°C, 30 s at 50°C, 1 min at 72°C for MLST and 1.5 min at 72°C for CRISPR-Cas type II loci, followed by a final elongation step of 7 min at 72°C. Primers used for MLST and detection of CRISPR-Cas type II loci are listed in Supplementary Table S2.

### PCR-based identification of CRISPR-Cas type II systems in *Streptococcus anginosus*

**Figure 1:**
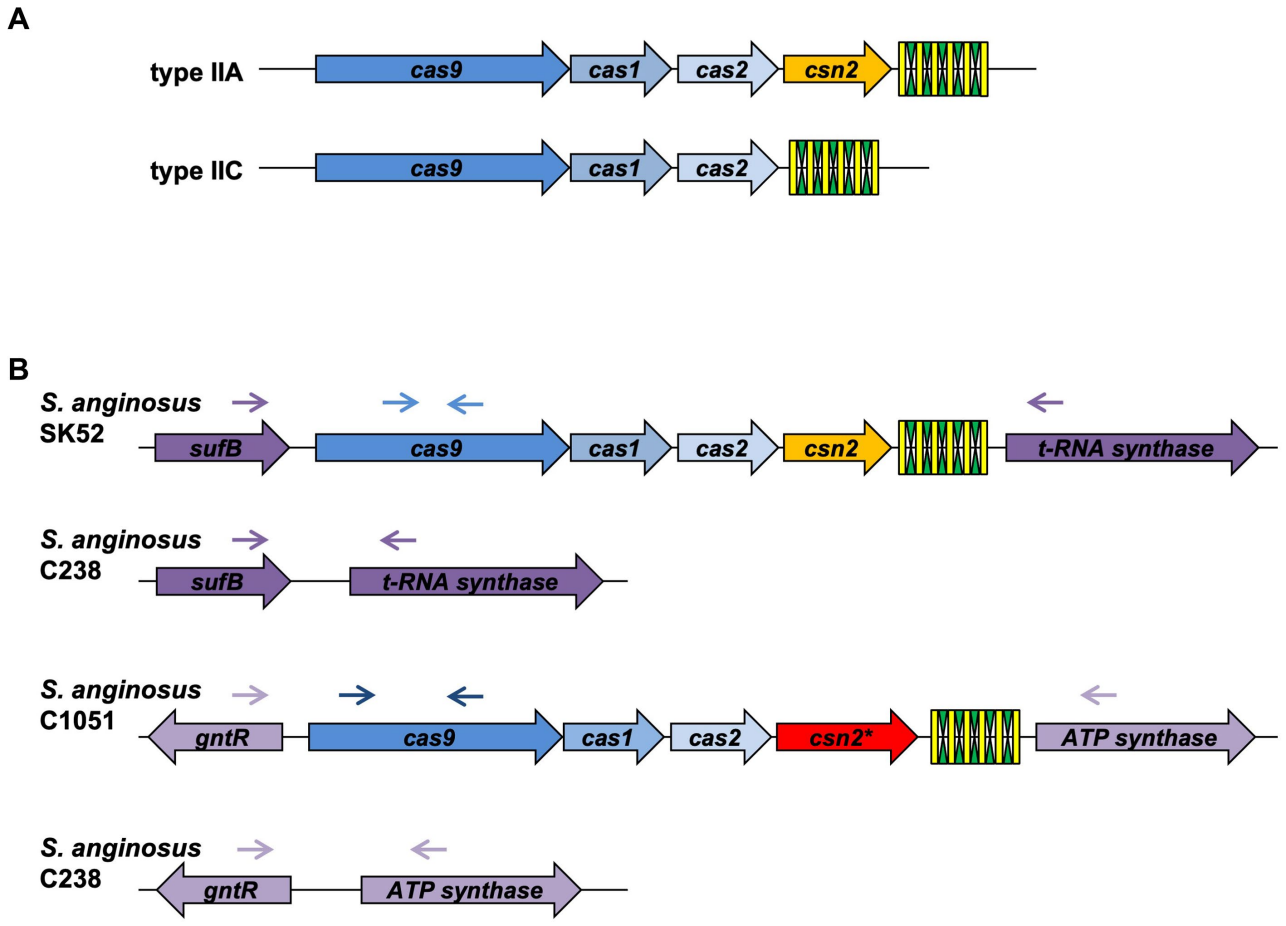
Genetic organization of CRISPR-Cas type II systems. **(A)** Conventional composition of CRISPR-Cas type II-A and II-C systems. **(B)** Genetic locus of CRISPR-Cas type II systems in *Streptococcus anginosus*. Green and yellow symbols represent repeat and spacer sequences of the CRISPR-Cas array. Genes present in all type II CRISPR-Cas systems are depicted in shades of blue, *Csn2* genes are shown in yellow for the canonical form and red for the long version. Lilac depicts genes, which are not part of the CRISPR-Cas locus. Arrows illustrate the location of primers used to screen for the presence and absence of CRISPR-Cas type II systems in *S. anginosus* isolates.

*Streptococcus anginosus* whole genome sequences were analyzed for CRISPR-Cas type II systems by the CRIPRS finder program located at the following website: https://crisprcas.i2bc.paris-saclay.fr/. In the publicly available genomes of *S. anginosus*, two CRISPR-Cas type II systems can be detected that are encoded in two different genomic locations of GenBank entry NC_022239.1 (CRISPR_A: SANR_RS04955, SANR_RS04950; CRISPR_B: SANR_RS07450, SANR_RS07440). The genes encoding Cas9 proteins in these two different CRISPR loci display nucleotide differences allowing a discrimination of the alleles by specific primers. The absence of a CRISPR locus in *S. anginosus* strains lacking a PCR product with primers targeting the two distinct CRISPR loci was confirmed through a set of primers adjacent to these typical CRISPR regions (SANR_RS04955, SANR_RS04950 and SANR_RS07450, SANR_RS07440). Primer sequences are listed in Supplementary Table S2, primer binding sites are depicted in Figure 1.

### Phylogenetic analysis and statistics

**Figure 2:**
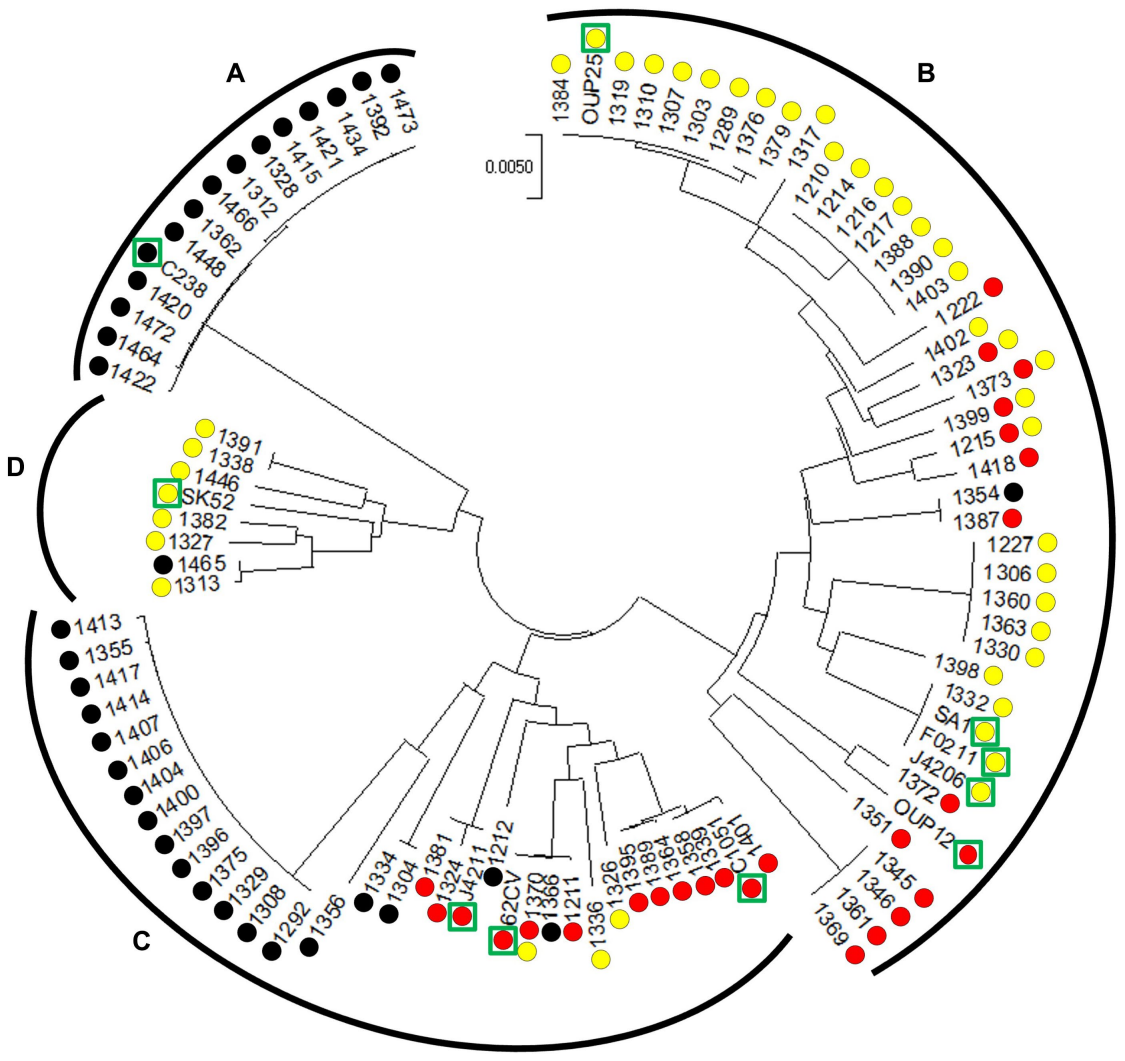
Circular minimal evolution tree constructed on the sequence of seven housekeeping genes illustrating the distribution of CRISPR-Cas type II systems in *S. anginosus* isolates. The units of the number of base substitutions per site are depicted. Subspecies [*S. anginosus* subsp. *whileyi* **(A)**, *S. anginosus* subsp. *anginosus* **(B)**] and genomosubspecies [*S. anginosus* genomosubsp. *AJ1* **(C)** *S. anginosus* genomosubsp. *Vellorensis* **(D)**] are highlighted. Circles represent presence and absence of CRISPR-Cas type II systems. Yellow: type II-A. Red: type II-C. Black: no type II system. Green rectangles highlight sequenced strains derived from GenBank.

The GenBank Database^1^ served as source for nucleotide and protein sequences and the Basic Local Alignment Search Tool (BLAST) was used to identify homologous sequences. To correctly assign *S. anginosus* subspecies and genomosubgroups as described by Jensen et al. (2013) *S. anginosus* strains were subjected to phylogenetic analysis based on MLST data (Figure 2). For MLST analysis the sequence of seven housekeeping genes (*map*, *pfl*, *ppaC*, *pyk*, *rpoB*, *sodA*, and *tuf*) was determined and aligned as previously described (Bishop et al., 2009). MEGA version 7 was then used for phylogenetic analysis of the obtained sequences (Kumar et al., 2018) by applying the Minimal Evolution method (Rzhetsky and Nei, 1993). Evolutionary distances were computed using the Maximum Composite Likelihood method (Tamura et al., 2004). The rate variation among sites was modeled with a gamma distribution (shape parameter = 20) and the minimum evolution tree was searched using the Close-Neighbor-Interchange (CNI) algorithm at a search level of 1. The initial tree was generated by using the Neighbor-joining algorithm (Saitou and Nei, 1987). The sequences of the seven housekeeping genes of *Streptococcus intermedius* (strain SV 101), *S. constellatus* (strain SV 019 and SV 100), *S. anginosus* (strain 557, 62CV, C1051, C238, F0211, J4206, J4211, OUP12, OUP25, SA1 and SK52) were retrieved from the GenBank database and served as reference in the analysis.

**Figure 3:**
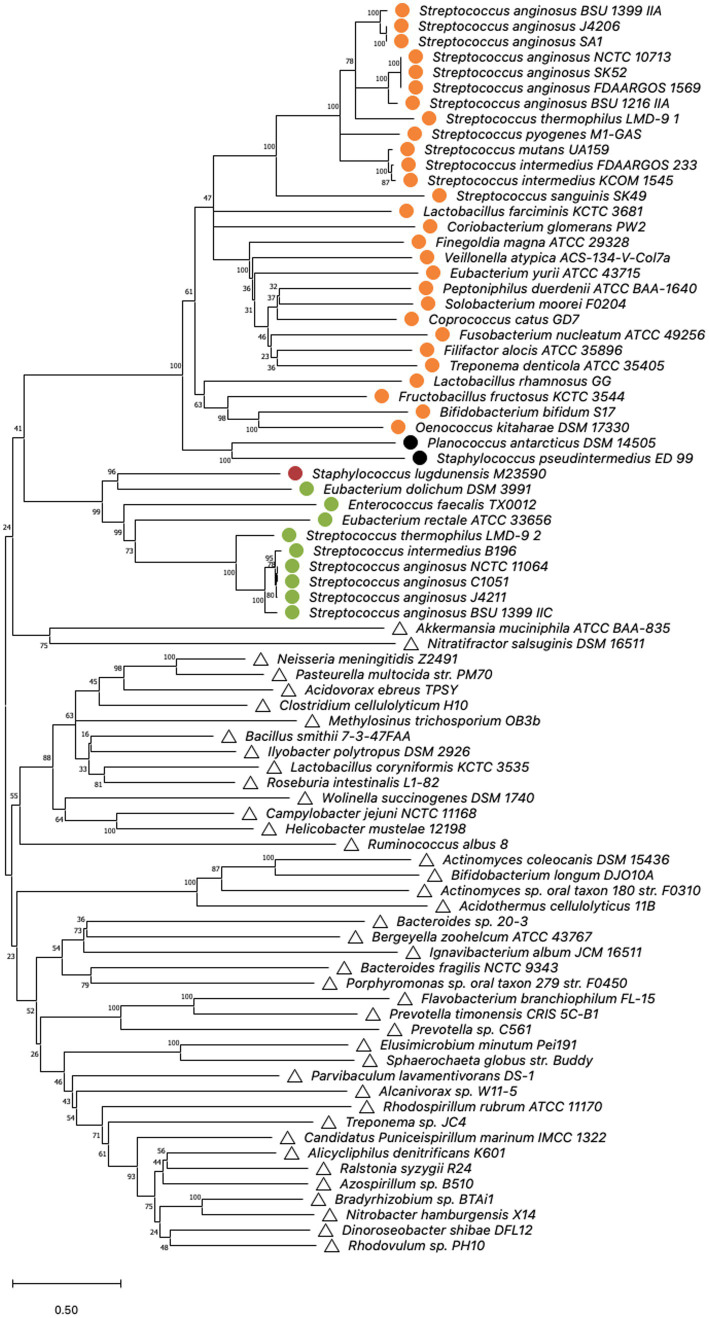
Cas9 phylogeny. Minimal Evolution tree constructed on the sequences of selected type IIA and IIC cas9 nucleases published by Chylinski et al. (2014); Fonfara et al. (2014) by bootstrap analysis using MEGA X. Multiple alignment of Cas9 amino acid sequences was constructed using the MUSCLE algorithm with default settings. The units of the number of amino acid substitutions per site are depicted. Type II-A systems are represented by circles, whereas type II-C systems are indicated by triangles. Corresponding *csn2* types stated by Chylinski et al. (2014) are highlighted. Orange: *csn2* type I, green: *csn2* type II, black: *csn2* type III and red: *csn2* type V.

To examine the phylogeny of *S. anginosus* Cas9 variants, sequences of selected type II-A and II-C Cas9 nucleases (Figure 3; Supplementary Table S3) as described previously in (Chylinski et al., 2014; Fonfara et al., 2014) were aligned using MUSCLE algorithm with default parameters. MEGA version X was used for bootstrap analysis (100 replicates) of obtained sequences (Felsenstein, 1985; Kumar et al., 2018; Stecher et al., 2020) and conducted applying Minimal Evolution method (Rzhetsky and Nei, 1993). The evolutionary distances were computed using the JTT matrix-based method (Jones et al., 1992) and are depicted in the units of the number of amino acid substitutions per site. Rate variation among sites was modeled with a gamma distribution (shape parameter = 20). The minimal evolution tree was searched using CNI algorithm at a search level of 1 (Nei and Kumar, 2000). The Neighbor-joining algorithm was used to generate the initial tree (Saitou and Nei, 1987). Analysis involved 81 amino acid sequences and all ambiguous positions were removed for each sequence pair (pairwise deletion option).

**Figure 4:**
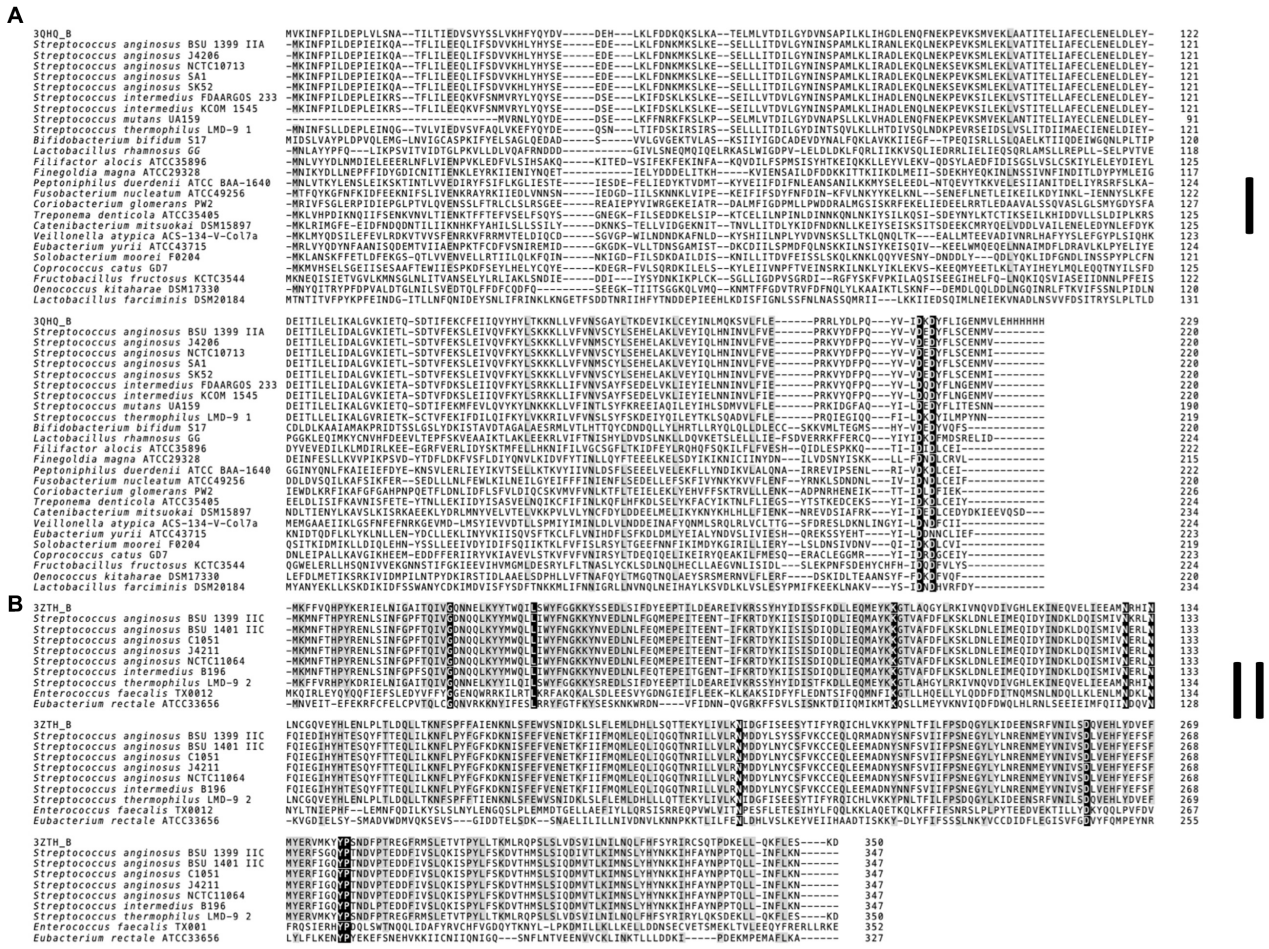
Multiple sequence alignment of Csn2 subfamilies found in *S. anginosus.* **(A)** Canonical 220 aa variant (Csn2 type I). **(B)** 350 aa long Csn2 variant (Csn2 type II). For each Csn2 subfamily the multiple sequence alignment of selected protein sequences was constructed separately using the MUSCLE algorithm with default settings. Homologs of each Csn2 subfamily were identified using HHPRED. Retrieved Csn2 sequences of *S. thermophilus* LMG 18311 (PDB: 3ZTH) and *S. agalactiae* ATCC 13813 (PDB: 3QHQ) were included. The positions strongly conserved (> 95% consensus) are shown by reverse shading, whereas above 70% consensus is highlighted in grey.

The multiple sequence alignment of the *S. anginosus* Csn2 variants (Figure 4) was constructed using MUSCLE algorithm for each Csn2 subfamily separately, with default settings. For each Csn2 subfamily, homologs were identified using HHPRED (Gabler et al., 2020) and obtained sequences *S. thermophilus* LMG 18311 (3ZTH) and *S. agalactiae* ATCC 13813 (3QHQ) were included.

GenBank entries of *S. anginosus cas9* and *csn2* newly obtained sequences can be accessed under the following numbers (OQ622068, OQ622069, OQ622070, OQ622071, OQ622072, OQ622073).

## Results

### Genomic organization of CRISPR-Cas in *Streptococcus anginosus*

Analyzing the CRISPR-Cas systems of whole genome sequences of *S. anginosus* strains available in GenBank and strains from our collection three different genotypes could be detected: Strains without any CRISPR arrays, isolates harboring one of two distinct CRISPR loci and strains harboring two different CRISPR arrays (Table 1). The most frequently found CRISPR locus displays the classical genomic organization of a CRIPSR-Cas type II-A system with *cas9* encoding the nuclease followed by *cas1*, *cas2* and *csn2* genes and the CRISPR array (Figure 1). In all of the analyzed *S. anginosus* strains this typical type II-A locus is incorporated between the *sufB* and a tRNA synthase gene. The second detected CRISPR locus also displays the canonical architecture of a CRISPR-Cas type II locus and is found between the *gntR* and an ATP synthase gene. At first glance it seems to be missing an easily identifiable *csn2* gene downstream of the *cas2* gene. However, closer analysis reveals that it contains a longer variant of a *csn2* gene in this genetic location. BLAST analysis of this *csn2* gene revealed closest homologies to the 350 amino acid (aa) long variant of the *csn2* gene of *Streptococcus thermophilus* LMG 18311 (42% identity and 65% similarity of the aa sequence; Figure 4). In each of the 27 strains containing this long variant of *csn2,* the corresponding CRISPR-Cas type II system was inserted downstream of *gntR*. CRISPR-Cas type I and CRISPR-Cas type III systems were not identified by the CRISPR finder program^2^ for the *S. anginosus* strains listed in Table 1.

**Table 1:** Clustered regularly interspaced short palindromic repeats (CRISPR) Cas system annotation of *Streptococcus anginosus* whole genome sequences.

Strain designation	CRISPR-Cas type II-A	CRISPR-Cas type II-C	none	# of spacers	Isolation source
*Streptococcus anginosus* BSU 1399	×	×		35	Soft tissue swab
*Streptococcus anginosus* C1051		×		29	Blood culture
*Streptococcus anginosus* C238			×	–	Respiratory tract
*Streptococcus anginosus* FDAARGOS_1155			×	–	Not available
*Streptococcus anginosus* FDAARGOS_1569	×			23	Not available
*Streptococcus anginosus* J4206	×			26	Blood culture
*Streptococcus anginosus* J4211		×		18	Hospital isolate
*Streptococcus anginosus* NCTC10713	×			23	Not available
*Streptococcus anginosus* NCTC11064		×		28	Materia alba tooth surface
*Streptococcus anginosus* SA1	×			35	Urine isolate
*Streptococcus anginosus* subsp. *whileyi* MAS624			×	–	Not available

### Phylogenetic analysis of *Streptococcus anginosus* strains

To analyze the population of *S. anginosus* in more detail and to investigate if strains carrying a specific CRISPR-Cas type II system cluster within specific subgroups of *S. anginosus,* a phylogenetic analysis based on housekeeping genes used for MLST analysis of viridans streptococci was conducted (Bishop et al., 2009). The analysis was carried out with the Mega7 program and included *S. anginosus* strains of clinical origin (Bauer et al., 2020) as well as whole genome sequences deposited in the GenBank database. The results showed a population structure representing the recognized subspecies and subgroups of *S. anginosus* as previously described (Jensen et al., 2013; Babbar et al., 2017; Figure 2). A correlation with the presence and absence of CRISPR-Cas type II systems demonstrated the absence of CRISPR-Cas in all 15 strains of *S. anginosus* subspecies *whileyi*, while all of the 43 *S. anginosus* subspecies *anginosus* strains carried either one or both of the previously described CRISPR-Cas type II systems. The 34 strains of the *S. anginosus* genomosubspecies AJ displayed the most diverse picture, while 19 strains had no CRISPR-Cas type II system, the remaining 15 strains harbored one of the two distinct CRISPR-Cas type II systems or both of them. In *S. anginosus* genomosubspecies *vellorensis* only the classical type II-A system could be detected in 7 of the 8 analyzed strains. In summary the strains harboring a CRISPR-Cas type II system, previously labeled as type II-C, did not cluster together within a certain subgroup of the *S. anginosus* strains.

### Phylogeny of Cas9 nucleases from CRISPR type II-A and CRISPR type II-C systems

The structures and sequences of Cas9 nucleases associated with CRISPR-Cas type II systems have previously been shown to be distinct for types II-A and II-C (Chylinski et al., 2014). To investigate if the Cas9 sequences of the two type II systems that are present in *S. anginosus* cluster either with type II-A or with type II-C a bootstrap analysis of available Cas9 sequences was performed using the MEGA X program (Figure 3). For comparison reasons, the analysis included Cas9 sequences from 39 different bacterial species including 18 streptococci carrying type II-A systems and 41 isolates from a variety of different bacterial species harboring CRISPR-Cas type II-C. All of the *S. anginosus* strains clustered together with the CRISPR type II-A Cas9 sequences, including the Cas9 sequences of strains reported to harbor a type II-C system. Also included in the analysis were Cas9 sequences from the GenBank database of *S. intermedius*, which belongs to the *S. anginosus* group. The bootstrap analysis located these sequences right next to the Cas9 sequences of *S. anginosus* strains within the CRISPR-Cas type II-A cluster, reflecting the close relationship between *S. intermedius* and *S. anginosus*. Interestingly none of the streptococcal sequences clustered in the vicinity of the Cas9 proteins of established CRISPR-Cas type II-C systems. In summary, the amino acid sequence analysis of available Cas9 proteins did not support the hypothesis that *S. anginosus* carries a CRISPR-Cas type II-C system.

## Discussion

CRISPR-Cas type II systems are frequently found in pathogenic bacteria (Grissa et al., 2007; Jensen et al., 2013; Louwen et al., 2014), comprising Gram negative as well as Gram positive species. In comparison to type I and III, the CRISPR-Cas type II systems are simple, harboring only two to three CRISPR associated (Cas) genes. The canonical type II-A system of *Streptococcus pyogenes* consists of the gene encoding the Cas9 nuclease followed by *cas1*, *cas2* and *csn2* genes (Jansen et al., 2002; Le Rhun et al., 2019; Figure 1). Cas1 and Cas2 are conserved within CRISPR-Cas systems of different species. They function as endonucleases and mediate the spacer acquisition step of the CRISPR-Cas system (Nunez et al., 2014). Csn2 also interacts with double stranded DNA and participates in spacer acquisition (Ellinger et al., 2012; Lee et al., 2012; Chylinski et al., 2014). But, while Csn2 is always present in type II-A systems, it is classically absent in CRISPR-Cas type II-C systems (Mir et al., 2018). Among pathogenic bacteria CRISPR-Cas type II-C systems are typically found in Campylobacter, Corynebacteria, Neisseria and Pasteurella species (Mir et al., 2018).

First genome projects showed the presence of typical CRISPR type II-A systems in *S. anginosus* strains (Olson et al., 2013), with several of the analyzed strains harboring more than one CRISPR array. Annotations in the CRISPR database (Grissa et al., 2007) show the typical CRISPR type II-A systems that have previously been described but also characterize several *S. anginosus* strains as harboring a CRISPR-Cas type II-C locus, since a typical *csn2* gene cannot readily be identified in the corresponding genomes (Gong et al., 2019; Lemaire et al., 2022). However, analyzing the CRISPR-Cas loci found in *S. anginosus* genomes that do not resemble the canonical type II-A system, we detected an additional gene downstream of *cas2*. Sequence comparison revealed significant homology to a Csn2 variant of *Streptococcus thermophilus* (Figure 4). This larger variant of Csn2, which has first been described in 2012 (Lee et al., 2012) is about 350 aa long and does not show any significant sequence homology to the canonical 220 aa long *csn2* gene of classical type II-A systems. The protein structure of this large Csn2 variant of *Streptococcus thermophilus* has been solved (Lee et al., 2012), showing a homotetradimer that forms a central channel, binding linear double stranded DNA presumably through the interaction with basic amino acids. Its precise function remains however unclear, since neither a nuclease nor integrase activity has been demonstrated in functional assays. The Csn2 protein appears to be crucial for CRISPR-Cas type II-A systems, while it has not been found in any other CRISPR systems (Makarova et al., 2020). It has previously been proposed that CRISPR-Cas type II-A systems developed from CRISPR-Cas type II-C systems through the acquisition of a *csn2* gene (Chylinski et al., 2014).

To evaluate if phylogenetic analysis supports the hypothesis that *S. anginosus* carries two different CRISPR-Cas type II-A systems we performed Cas9 phylogeny. Cas9 phylogeny has previously been shown to allow a differentiation between different CRISPR-Cas systems (Chylinski et al., 2014). Comparison of the Cas9 sequences of *S. anginosus* present in the two distinct CRISPR-Cas type II systems with Cas9 sequences available in GenBank, showed a clustering of both *S. anginosus* Cas9 nucleases with Cas9 enzymes of other CRISPR-Cas type II-A systems (Figure 3). The results further demonstrate a separate clustering of the Cas9 nucleases of CRISPR-Cas type II-C systems. None of the *S. anginosus* Cas9 sequences can be found in the type II-C clusters. Furthermore, an MLST analysis of multiple *S. anginosus* strains was performed to investigate, if the two distinct CRISPR-Cas type II systems associate with different *S. anginosus* subspecies or *S. anginosus* genomosubgroups (Figure 2). As observed in previous studies (Jensen et al., 2013; Babbar et al., 2017), known subspecies and genomosubgroups of the species are clearly identifiable. However, similar to the results of the Cas9 phylogeny, no indication of a clear separation of strains carrying one of the two type II systems can be detected.

With our enlarging knowledge about CRISPR-Cas systems it has become clear that besides their function in bacterial immunity CRISPR-Cas plays a role in virulence (Louwen et al., 2014) and may also have additional functions (Makarova et al., 2020). The association of CRISPR-Cas with virulence was first detected in *Francisella novicida*, where Cas9 inhibits transcription of a lipoprotein as an immune evasion mechanism (Weiss et al., 2007; Ratner et al., 2019). In streptococci several examples exist for the association of virulence and CRISPR-Cas. In *Streptococcus agalactiae* the highly virulent genetic lineage ST17 carries a different CRISPR-Cas profile (Lier et al., 2015). *Streptococcus mutans* strains carrying CRISPR-Cas display an increased biofilm formation and exopolysaccharide production, which is crucial for their virulence (Chen et al., 2017). In regard to hemolysin genes of *S. anginosus* we were able to show that the presence of *sag* genes is associated with the absence of CRISPR-Cas systems (Bauer et al., 2020). Increasing our knowledge about the diversity of *S. anginosus* CRISPR-Cas systems may thus also play a role in assessing the virulence potential of *S. anginosus* strains.

Taken together our data do not support the existence of a separate CRISPR Cas type II-C system in the *S. anginosus* strains we analyzed. In contrast to the currently used classifications a *csn2* variant could be detected in type II systems not fitting into the classical CRISPR-Cas type II-A structure. Phylogenetic analysis supports the hypothesis that these strains carry a variant CRISPR-Cas type II-A system.

## Data availability statement

The datasets presented in this study can be found in online repositories. The names of the repository/repositories and accession number(s) can be found at: https://www.ncbi.nlm.nih.gov/genbank/, OQ622068, OQ622069, OQ622070, OQ622071, OQ622072, and OQ622073.

## Funding

The work of RB and DH was supported through the Bausteine Program of the Medical Faculty, University of Ulm and the International Graduate School in Molecular Medicine Ulm.

## Author contributions

BS and RB designed the study. BS, RB, and DH wrote the manuscript. RB, DH, AG, RR, and SM performed experiments and analyzed the data. AG, RR, and SM edited and modified the manuscript. All authors contributed to the article and approved the submitted version.

## Conflict of interest

The authors declare that the research was conducted in the absence of any commercial or financial relationships that could be construed as a potential conflict of interest.

## Publisher’s note

All claims expressed in this article are solely those of the authors and do not necessarily represent those of their affiliated organizations, or those of the publisher, the editors and the reviewers. Any product that may be evaluated in this article, or claim that may be made by its manufacturer, is not guaranteed or endorsed by the publisher.

## Footnotes

^1^ http://www.ncbi.nlm.nih.gov/

^2^ https://crisprcas.i2bc.paris-saclay.fr/
