# Supplementary material for: Diversity of CRISPR-Cas type II-A systems in Streptococcus anginosus
Source: Front Microbiol. 2023 Jun 15;14:1188671. doi: 10.3389/fmicb.2023.1188671 (PMC10310304; doi:10.3389/fmicb.2023.1188671)
Supplement: Supplementary file 1 [file Table_1.DOCX]

**Supplemental information**

Table S1: Bacterial strain collection

| Material | Strain | Source |
| --- | --- | --- |
| Human throat | *S. anginosus* type strain SK52, ATCC33397, BSU 458^+^, | ATCC |
| Blood culture | BSU 1210, BSU 1211, BSU 1212, BSU 1216, BSU 1217, BSU 1222, BSU 1227, BSU 1384, BSU 1466 | Ulm/Aachen collection |
| Bone swab | BSU 1339, BSU 1344 | Ulm/Aachen collection |
| Gastrointestinal abscess | BSU 1306, BSU 1308, BSU 1323, BSU 1328, BSU 1331, BSU 1332, BSU 1354, BSU 1356, BSU 1358, BSU 1369, BSU 1388, BSU 1412, BSU 1421, BSU 1446 | Ulm/Aachen collection |
| Mucosal membrane | BSU 1312, BSU 1313, BSU 1396, BSU 1397, BSU 1404, BSU 1415, BSU 1434, BSU 1447, BSU 1448, BSU 1464, BSU 1465, BSU 1472, BSU 1473 | Ulm/Aachen collection |
| Soft tissue swab | BSU 1289, BSU 1292, BSU 1303, BSU 1304, BSU 1307, BSU 1318, BSU 1324, BSU 1326, BSU 1327, BSU 1329, BSU 1330, BSU 1334, BSU 1336, BSU 1338, BSU 1351, BSU 1355, BSU 1360, BSU 1362, BSU 1363, BSU 1366, BSU 1372, BSU 1373, BSU 1375, BSU 1376, BSU 1379, BSU 1381, BSU 1382, BSU 1386, BSU 1387, BSU 1389, BSU 1391, BSU1392^+^, BSU 1395, BSU 1398, BSU 1399, BSU 1400, BSU 1401, BSU 1402, BSU 1403, BSU 1405, BSU 1406, BSU 1407, BSU 1413, BSU 1417, BSU 1418, BSU 1419, BSU 1420, BSU 1422 | Ulm/Aachen collection |
| Urine isolate | BSU 1414 | Ulm/Aachen collection |

Table S2: PCR primers used in this study

| Gene/locus | Primer | Sequence (5ʹ→3ʹ( | Reference |
| --- | --- | --- | --- |
| *map* (MLST) | map_up | GCWGACTCWTGTTGGGCWTATGC | ([Bishop *et al.*, 2009](#_ENREF_5)) |
|  | map_down | TTARTAAGTTCYTTCTTCDCCTTG |  |
| *pfl* (MLST) | pfl_up | AACGTTGCTTACTCTAAACAAACTGG | ([Bishop *et al.*, 2009](#_ENREF_5)) |
|  | pfl_down | ACTTCRTGGAAGACACGTTGWGTC |  |
| *ppaC* (MLST) | ppaC_up | GACCAYAATGAATTYCARCAATC | ([Bishop *et al.*, 2009](#_ENREF_5)) |
|  | ppaC_down | TGAGGNACMACTTGTTTSTTACG |  |
| *pyk* (MLST) | pyk_up | GCGGTWGAAWTCCGTGGTG | ([Bishop *et al.*, 2009](#_ENREF_5)) |
|  | pyk_down | GCAAGWGCTGGGAAAGGAAT |  |
| *rpoB* (MLST) | rpoB_up | AARYTIGGMCCTGAAGAAAT | ([Bishop *et al.*, 2009](#_ENREF_5)) |
|  | rpoB_down | TGIARTTTRTCATCAACCATGTG |  |
| *sodA* (MLST) | sodA_up | TRCAYCATGAYAARCACCAT | ([Bishop *et al.*, 2009](#_ENREF_5)) |
|  | sodA_down | ARRTARTAMGCRTGYTCCCARACRTC |  |
| *tuf* (MLST) | tuf_up | GTTGAAATGGAAATCCGTGACC | ([Bishop *et al.*, 2009](#_ENREF_5)) |
|  | tuf_down | GTTGAAGAATGGAGTGTGACG |  |
| CRISPR_A | A_CRISPR_Cas9_fwd | TTAAACGGCGTGGTATCAGC | This study |
|  | A_CRISPR_Cas9_rev | ATTCCTGCGCGGTATAAGAC |  |
| CRISPR_A negative | A_CRISPR_control_fwd | GCCTGAAATAATAGTGGTTG | This study |
|  | A_CRISPR_control_rev | GGTTTTATCATCTTAACTGTC |  |
| CRISPR_B | B_CRISPR_Cas9_fwd | GGCAGAATATAAGGCGGATG | This study |
|  | B_CRISPR_Cas9_rev | AACCATCATCGATTAGATAG |  |
| CRISPR_B negative | B_CRISPR_control_fwd | AACAGCGCCATCTGGGAAAG | This study |
|  | B_CRISPR_control_rev | GGTATTAGCGAAGAAGAAGC |  |

Table S3: Bacterial strains used for phylogenetic analysis of Cas9 and multiple sequence alignment of Csn2. Protein sequences are denoted by Genbank identifiers (GI) and complete organism name. The nucleotide sequences of strains of our collection are indicated by their GenBank accession number.

| Cas9 sequences | Csn2 sequences | Complete strain name |
| --- | --- | --- |
| 117929158 | - | *Acidothermus cellulolyticus* 11B |
| 222109285 | *-* | *Acidovorax ebreus* TPSY |
| 227494853 | *-* | *Actinomyces coleocanis* DSM 15436 |
| 315605738 | *-* | *Actinomyces* sp. oral taxon 180 str. F0310 |
| 187736489 | *-* | *Akkermansia muciniphila* ATCC BAA-835 |
| 407803669 | *-* | *Alcanivorax* sp. W11-5 |
| 330822845 | *-* | *Alicycliphilus denitrificans* K601 |
| 288957741 | *-* | *Azospirillum* sp. B510 |
| 365156657 | *-* | *Bacillus smithii* 7-3-47FAA |
| 60683389 | *-* | *Bacteroides fragilis* NCTC 9343 |
| 301311869 | *-* | *Bacteroides* sp. 20-3 |
| 423317190 | *-* | *Bergeyella zoohelcum* ATCC 43767 |
| 310286728 | 310286726 | *Bifidobacterium bifidum* S17 |
| 189440764 | *-* | *Bifidobacterium longum* DJO10A |
| 148255343 | *-* | *Bradyrhizobium* sp. BTAi1 |
| 218563121 | *-* | *Campylobacter jejuni* NCTC 11168 |
| 294086111 | *-* | *Candidatus Puniceispirillum marinum* IMCC 1322 |
| - | 224543315 | *Catenibacterium mitsuokai* DSM 15897 |
| 220930482 | *-* | *Clostridium cellulolyticum* H10 |
| 291520705 | 291520707 | *Coprococcus catus* GD7 |
| 328956315 | 328956318 | *Coriobacterium glomerans* PW2 |
| 159042956 | *-* | *Dinoroseobacter shibae* DFL12 |
| 187250660 | *-* | *Elusimicrobium minutum* Pei191 |
| 315149830 | 422729713 | *Enterococcus faecalis* TX0012 |
| 160915782 | *-* | *Eubacterium dolichum* DSM 3991 |
| 238924075 | 238924078 | *Eubacterium rectale* ATCC 33656 |
| 306821691 | 306821688 | *Eubacterium yurii* ATCC 43715 |
| 374307738 | 374307735 | *Filifactor alocis* ATCC 35896 |
| 169823755 | 169823758 | *Finegoldia magna* ATCC 29328 |
| 347536497 | *-* | *Flavobacterium branchiophilum* FL-15 |
| 339625081 | 339625078 | *Fructobacillus fructosus* KCTC 3544 |
| 34762592 | 34762595 | *Fusobacterium nucleatum* ATCC 49256 |
| 291276265 | *-* | *Helicobacter mustelae* 12198 |
| 385811609 | *-* | *Ignavibacterium album* JCM 16511 |
| 310780384 | *-* | *Ilyobacter polytropus* DSM 2926 |
| 336393381 | *-* | *Lactobacillus coryniformis* KCTC 3535 |
| 336394882 | 336394885 | *Lactobacillus farciminis* KCTC 3681 |
| 258509199 | 258509196 | *Lactobacillus rhamnosus* GG |
| 296446027 | *-* | *Methylosinus trichosporium* OB3b |
| 218767588 | *-* | *Neisseria meningitidis* Z2491 |
| 319957206 | *-* | *Nitratifractor salsuginis* DSM 16511 |
| 92109262 | *-* | *Nitrobacter hamburgensis* X14 |
| 366983953 | 372325148 | *Oenococcus kitaharae* DSM 17330 |
| 154250555 | *-* | *Parvibaculum lavamentivorans* DS-1 |
| 15602992 | *-* | *Pasteurella multocida* str. Pm70 |
| 304438954 | 304438951 | *Peptoniphilus duerdenii* ATCC BAA-1640 |
| 389815359 | *-* | *Planococcus antarcticus* DSM 14505 |
| 402847315 | *-* | *Porphyromonas* sp. oral taxon 279 str. F0450 |
| 345885718 | *-* | *Prevotella* sp. C561 |
| 282880052 | *-* | *Prevotella timonensis* CRIS 5C-B1 |
| 344171927 | *-* | *Ralstonia syzygii* R24 |
| 83591793 | *-* | *Rhodospirillum rubrum* ATCC 11170 |
| 402849997 | *-* | *Rhodovulum* sp. PH10 |
| 225377804 | *-* | *Roseburia intestinalis* L1-82 |
| 325677756 | *-* | *Ruminococcus albus* 8 |
| 320528778 | 493573666 | *Solobacterium moorei* F0204 |
| 325972003 | *-* | *Sphaerochaeta globus* str. Buddy |
| 315659848 | *-* | *Staphylococcus lugdunensis* M23590 |
| 323463801 | *-* | *Staphylococcus pseudintermedius* ED 99 |
| - | 374414489 | *Streptococcus agalactiae* ATCC 13813 |
| OQ622068 | *-* | *Streptococcus anginosus* BSU 1216 |
| OQ622069  OQ622070 | OQ622071  OQ622072 | *Streptococcus anginosus* BSU 1399 |
| - | OQ622073 | *Streptococcus anginosus* BSU 1401 |
| 537634371 | 537634374 | *Streptococcus anginosus* C1051 |
| 1049060090 | 1049060087 | *Streptococcus anginosus* J4206 |
| 941941216 | 941941219 | *Streptococcus anginosus* J4211 |
| 1539733176 | 1539733175 | *Streptococcus anginosus* NCTC 10713 |
| 1654229872 | 1654229872 | *Streptococcus anginosus* NCTC 11064 |
| 674114972 | 674114975 | *Streptococcus anginosus* SA1 |
| 1178154693 | 333767645 | *Streptococcus anginosus* SK52 |
| 2127629375 | *-* | *Streptococcus anginosus* subsp. *anginosus* FDAARGOS_1569 |
| 537628848 | 537628851 | *Streptococcus intermedius* B196 |
| 1169387830 | 1169388055 | *Streptococcus intermedius* FDAARGOS_233 |
| 929734708 | 929736043 | *Streptococcus intermedius* KCOM_1545 |
| 24379809 | 488192338 | *Streptococcus mutans* UA159 |
| 13622193 | - | *Streptococcus pyogenes* M1 GAS |
| 422884106 | *-* | *Streptococcus sanguinis* SK49 |
| 116628213 | 116101487 | *Streptococcus thermophilus* LMD-9 1 |
| 116627542 | 116100822 | *Streptococcus thermophilus* LMD-9 2 |
| - | 394986200 | *Streptococcus thermophilus* LMG 18311 |
| 42525843 | 42525846 | *Treponema denticola* ATCC 35405 |
| 384109266 | *-* | *Treponema* sp. JC4 |
| 303229466 | 303229343 | *Veillonella atypica* ACS-134-V-Col7a |
| 34557790 | *-* | *Wolinella succinogenes*_DSM 1740 |
